# Understanding what shapes patient and family experience of the open disclosure process in the Irish healthcare context: a qualitative study

**DOI:** 10.1186/s12910-026-01436-0

**Published:** 2026-03-11

**Authors:** Aoife De Brún, Eilish McAuliffe, Lorraine Schwanberg, Éidín Ní Shé

**Affiliations:** 1 UCD Centre for Interdisciplinary Research, Education, and Innovation in Health Systems at University College Dublin (UCD IRIS Centre), School of Nursing, Midwifery and Health Systems, University College Dublin, Dublin, Ireland; 2 Incident Management, National Quality and Patient Safety, Health Service Executive, Dublin, Ireland; 3 Graduate School Healthcare Management, Royal College of Surgeons Ireland, Dublin, Ireland

**Keywords:** Open disclosure, Qualitative research, Patient experiences, Patient safety incidents, Communication

## Abstract

**Background:**

When something goes wrong in healthcare and results in harm or potential harm to the patient, there is an ethical and moral duty for healthcare professionals to disclose the incident to the patient, their family or their relevant others.

**Objective:**

This study sought to explore experiences of open disclosure and identify aspects of the process that shaped patient/family experience.

**Design:**

This research was a retrospective qualitative descriptive study, interviewing those with recent experience of open disclosure.

**Setting and participants:**

In total, 6 patients/family members, 5 healthcare professionals and 5 patient advocates from Ireland took part in semi-structured interviews.

**Results:**

The results are organised into five themes: (i) The necessity of acknowledgement and apology, (ii) Importance of clear, compassionate and respectful communication, (iii) Openness and sharing as a two-way process, (iv) Healthcare staff as patients in open disclosure, and (v) The lasting impact of poor experiences of open disclosure. Participants emphasised the importance of apology, clear and compassionate communication and the value of creating space during meetings to listen to the experiences of those who have been impacted. It was reported there is a reluctance to use the term ‘open disclosure’ when discussing issues with patients, families and advocates. This may be due to a fear of litigation, fear that being associated with open disclosure means healthcare staff have made a mistake, and/or the existence of a blame culture within organisations. This research also highlighted the experiences of healthcare staff who themselves experience harm. Their experience when colleagues failed to be transparent was described as akin to a double trauma by participants.

**Conclusion:**

Participants emphasised key aspects of the process that shaped experience, including quality of communication, openness and the importance of apology and space for sharing experiences. It is evident that compounded harm is happening through poor understanding and implementation of open disclosure, and this is avoidable. Regular feedback from patients and families is important to improve processes and staff training and to ensure the process is meeting the needs of all those involved.

**Patient or public contribution:**

Patients, patient advocates and experts by experience (those with direct experience of open disclosure) were represented on the steering committee, which guided every phase of this research. They supported the research by providing feedback on study design and recruitment planning, supported sensitive recruitment, helped design the interview guide and reviewed and provided feedback on patient information materials and content forms.

**Supplementary Information:**

The online version contains supplementary material available at 10.1186/s12910-026-01436-0.

## Introduction

When something goes wrong in healthcare and results in harm or potential harm to the patient, there is an ethical and moral imperative on healthcare professionals to disclose the harm to patients (or their family or relevant others). This process of open and honest disclosure promotes timely and transparent communication with patients and families. Such practices are now recognised and embedded in policies in many health systems [1]. A growing body of research demonstrates that when an incident occurs, what is important to patients and families is a prompt disclosure, support from healthcare staff and organisations following a harmful patient safety incident [2, 3], along with the initiation of a clear disclosure process [4–9]. Open disclosure has the potential to benefit both the patients who have experienced harm and the healthcare staff involved in the incident [10]. 

Research on patient and family experiences has found that there can be a difference between patients and healthcare professionals regarding expectations and attitudes towards open disclosure [11]. Despite the evidence for patient and family expectations of honest and timely open disclosure, it does not always happen when it should and it does not always meet the needs and expectations of patients and families [12, 13]. Birks [14] describes the ‘disclosure gap’ as the notion of open disclosure being a moral imperative as compared to the reality of practice. Barriers to healthcare professionals engaging in open disclosure include fear of litigation, a culture of blame and a lack of confidence and/or a lack of skills or knowledge in how to communicate effectively when a patient safety incident occurs [11, 14, 15]. Service and policy-level issues, such as the alignment between open disclosure and incident investigation, may also influence the practice of open disclosure [16].

Patients’ experience is often shaped through effective communication. This includes receipt of an honest and sincere apology, being treated with empathy and respect, supporting patients and families to share their story, ask questions and to learn more about the incident that led to harm [17]. However, when open disclosure is poorly implemented, this can lead to further harm, or what is referred to as ‘compounded harm’. Researchers assert that compounded harm, which results when people feel unheard or dismissed, “can feel worse than the original injury” [18]. Poor open disclosure can lead to further harm to patients including feelings of anger, confusion, and a loss of trust in the healthcare system [10]. According to a recent review, the researchers concluded that despite the research conducted over more than two decades, compounded harm is still happening through poor implementation of open disclosure, and this is avoidable [17]. Examples of instances where open disclosure does not meet patient and family needs includes disclosure not being followed up, that is, no contact with the patient and family after an initial conversation about harm, staff not offering an apology, lack of timely disclosure, or the discussion not meeting the needs of affected parties [6].

Despite recent attention to the topic of open disclosure, there has been more emphasis on healthcare staff experiences and a relative lack of research on patient experiences, with some notable exceptions [2, 12, 13, 19–21]. Furthermore, when research has explored healthcare staff perspectives, clinicians and nurses tend to be the focus of these studies and thus, there has been a dearth of attention paid to the experiences of other healthcare team members and to patient advocates [22, 23]. This may be due to the challenges in talking to healthcare staff and patients about these distressing experiences, patients not wanting to relive their experiences or potential complications due to on-going investigations or legal proceedings [13]. However, such research is crucial so that we can learn from what might be working well or not so well and ensure that the process is meeting the needs of patients and families.

### Open disclosure in the Irish context

A policy of open disclosure was first implemented in Ireland in 2013. However, following a series of high-profile harmful patient safety incidents and a concomitant lack of openness from health services, there has been increased public attention on the topic and process of open disclosure. One area of learning in relation to open disclosure was Dr Gabriel Scally’s review of issues related to CervicalCheck, the Irish national cervical cancer screening programme, in 2015 [24]. The Scally report was published following a controversial audit of the screening programme, which focused public attention on the issue of open disclosure. Key recommendations from the investigation and report into this matter included revisions to the Open Disclosure policy to ensure patient access to their medical records, mandatory training for healthcare staff in open disclosure, and a framework for that includes evaluation and audit [24]. Additionally, the resulting report called for a “statutory duty of candour” to be on healthcare professionals and on the organisations for which they work [24]. This means a legal requirement to be open and honest with patients when safety incidents occur was recommended.

Given the focus on open disclosure in the Irish health system in recent years, this research sought to explore experiences of open disclosure from the perspective of those involved in patient safety incidents and what aspects of the process people felt shaped patient/family experience of the process. This research was part of a larger body of worked aimed at co-designing a tool to measure patient experience of the open disclosure process.

## Methods

### Study design

This was a retrospective descriptive qualitative study that explored the factors and aspects of the open disclosure (OD) process where experience is shaped. The study sought participation from patients and families with experience of the process, healthcare staff who have been involved and patient advocates who have supported patients and their relevant others through open disclosure. In-depth semi-structured interviews were conducted and explored people’s experience of a specific incident and/or their reflections if they had been involved or supported others through multiple open disclosures. Interview questions included topics related to the key steps in the process and queried participants’ perspectives on timeliness, the communication process, offers of apology and support, perception as to whether learning occurred as a result of the incident and what they felt worked particularly well or not so well and why (see supplemental file for interview guides). Interviews were conducted in-person or via Zoom, depending on the preference of the participant. Informed consent was obtained prior to interview and interviews were audio-recorded. Transcription was conducted by a third-party company through a data sharing and data processing agreement, with the provider bound by confidentiality. The researchers were experienced qualitative researchers and had no prior knowledge of the participants.

### Ethical considerations and ethical approval

Ethical issues considered in the planning of this research including the potential for participant distress recalling their experiences. Patient/public involvement (PPI) input through an advisory group guided the research team on sensitive recruitment and supported the identification of gatekeepers who knew potential participants and could identify those that may be suitable. A distress protocol was prepared to support participants in case they became upset and details of support organisation were shared with all participants after interviews. Participant confidentiality is crucial when researching such a sensitive topic and for that reason, we are not reporting any details of the incidents that led to OD due to the potential to identify the participants from these incidents.

### Participant recruitment

In the initial phase we recruited participants via collaborating partners and gatekeepers in the health system. Gatekeepers were regional and organisation OD Leads and project advisors with a role in patient advocacy or patient support groups. They were asked to identify people (patients/families, healthcare staff, patient advocates) who had recent experience of OD. The inclusion criteria were also shared with gatekeepers. Prospective participants were contacted with information packs and the consent form and asked to contact the research team directly if they had any questions or wanted to take part in the research.

#### Inclusion criteria

In this qualitative study, we hoped to provide further insight into the national context to inform how best to measure experience and to understand what is important to patients. Therefore, we sought to obtain a variety of perspectives on this and included patients/relevant persons^1^, healthcare staff and patient advocates in our sample. Invitations to participants were shared through gatekeepers. These included:

- (i)  people in patient support/advocacy roles who had worked with patients/relevant persons and supported them through OD processes,
- (ii)  OD Leads in healthcare settings, who identified staff and patients/relevant others with recent experience of OD.

Table 1 details the inclusion criteria for each participant group.

**Table 1:** Inclusion and exclusion criteria

Participant group	Inclusion criteria	Exclusion criteria	Target
Patients/relevant others	• Over 18 with capacity to provide informed consent • Experience of the OD process and have completed process • Not currently an in-patient in an acute healthcare setting • Incident occurred at least 6 months ago but within previous 3 years (*to ensure sensitivity in approach to prospective participants*) • Patient/service user has knowledge that an incident has occurred, and that OD has taken place.	• In an in-patient setting / vulnerable • Has not completed OD process • Incident occurred more than 3 years ago	6–10 participants
Healthcare staff	• Over 18 with capacity to provide informed consent • Have with experience of the OD process in the past 2 years • Have been those involved directly with incident and/or those who have supported patients/relevant others through the process	• No experience of the OD process • Has not supported patients/relevant others or staff through the process	5–7 participants
Patient advocates	• Over 18 with capacity to provide informed consent • Have experience of supporting patients/relevant others through the process in the past 2 years	• No experience of the OD process • Has not supported patients/relevant others through the process	3–5 participants

### Data collection and participant protection processes

Given the highly sensitive and personal nature of the topic, interviews were conducted by researchers with extensive expertise in qualitative interviewing. Details regarding psychological support services that were available to participants were shared with all participants after the event, in the event that they experienced distress recalling their experiences of OD. Following interviews, no participants demonstrated undue distress and the distress protocol [25–27] prepared for this purpose was not utilised.

### Data analysis

Transcripts were checked and verified, and any potentially identifiable information was removed to ensure confidentiality. All participants were offered the opportunity to review their transcript prior to it being included in the analysis. Two participants did this and made very minor changes (e.g., adding in a word), mostly to enhance clarity of their meaning.

Data were analysed using an inductive thematic approach [28]. This is a theoretically flexible approach to identifying themes and patterns in the data. Analysis followed the steps outlined by Braun and Clarke [28] where Step 1 involves familiarisation with the data and re-reading transcripts. In Step 2, initial codes were generated and this was supported by NVivo data management software [29]. Step 3 involved collating codes and searching for themes, Step 4 was reviewing and refining themes, Step 5 defining and naming themes and Step 6 writing up the findings. Adherences to this approach helped ensure codes were grounded in the data and that the trends observed in the data were captured as major themes. Primary data coding was conducted by the lead researcher (ADB), with a second researcher (ÉNS) independently coding a subset of manuscripts to support discussion and verification of coding and themes. The research team reviewed results regularly as the analysis progressed and discussed findings to challenge assumptions and to reach agreement on the final organisation of codes and themes.

### Reflexivity statements

The lead researcher (ADB) has a background in psychology and has been conducting qualitative healthcare research for more than 15 years. She has no personal experience of open disclosure but has supported family members in navigating the health system. She has previously worked with the co-researchers on this project but had no pre-existing relationships with any participants in this research. She had experience of conducting sensitive research and along with other team members was cognisant of ensuring that most sensitive and supportive approach was adopted in recruitment and data collection for this study. Maintaining and protecting participants and participant confidentiality has been a key consideration in this work and how we have presented the findings.

The second coder (ÉNS) has a disciplinary background in qualitative social science and has been engaged in health‑related research for over 15 years. While she did not have direct personal experience of OD she understands the broader systemic and interpersonal challenges faced by individuals navigating OD within healthcare contexts. She has experience in conducting sensitive and ethically complex interviews and throughout the study, she remained attentive to the potential influence of her own assumptions, disciplinary training, and positionality on the interpretation of participant accounts. Ensuring participant safety, emotional well-being, and confidentiality was a central concern that shaped both data collection and analysis. These considerations also informed how the findings have been represented, with attention to protecting participant identities.

## Results

### Sample descriptives

In total, 6 patient/family member interviews (all female participants) were conducted with those who had direct experience of OD, 5 interviews were conducted with healthcare professionals with direct experience of OD (1 male, 4 female) and 5 interviews were conducted with patient advocates or those in patient support roles (1 male, 4 female). The study invitation materials were shared also with five other potential patient participants who declined to take part. Interviews were conducted between September 2023 and January 2024 either in-person, by phone or remotely online via Zoom. Interviews lasted 49 min on average, with interview duration ranging from 35 min to 65 min.

Table 2 details further information about participants. In this section, participants are identified by their group with P representing patient, F representing a family member of a patient who has experienced harm, A representing a patient advocate and HCP indicating a quote from a healthcare professional.

**Table 2:** Participant details

Participant code	Speciality (area incident occurred or profession if HCP)	Incident Category as defined the HSE Incident Management Framework* [30]
P1	Maternity	Category 1
P2	Screening services	Category 1
P3	Emergency	Category 3
P4	Screening services	Category 1
F1	Emergency/radiology	Category 2
F2	Medication error	Category 1
HCP1	Profession: nursing	Multiple (across all categories)
HCP2	Profession: quality and patient safety management (acute setting)	Multiple (across all categories)
HCP3	Profession: quality and patient safety management (community setting)	Multiple (across all categories)
HCP4	Profession: quality and patient safety management (acute setting)	Multiple (across all categories)
HCP5	Profession: medicine	Multiple (across all categories)
A1-A5	Patient advocates	Multiple (across all categories)

*These categorisations are based on the research team’s categorisation of harm in line with the Incident Management Framework. The research team had no access to the incident or investigation reports that may be relevant to these events. The categorisation here is intended to convey the severity of harm experienced and the impact of the patient safety incident of the patient involved.

### Qualitative findings

While the participant stories were complex, all stories and experiences were instructive in highlighting aspects of the process where patient/family experiences were shaped. The results are organised into five themes: (i) The necessity of acknowledgement and apology, (ii) Importance of clear, compassionate and respectful communication, (iii) Openness and sharing as a two-way process, (iv) Healthcare staff as patients in OD, and (v) The lasting impact of poor experiences of OD.

#### The necessity of acknowledgement and apology

Participants recognised the need to have acknowledgement of what had happened to them. All interviewed agreed this was crucial, and as one advocate reflected, the majority of people who experience harm just want that basic acknowledgement.

“I would say near 100% of people that I’ve dealt with in this service, what they’re looking for is an acknowledgement of what has happened. And generally looking also for you know a sincere apology. Not a ‘we’re sorry you felt…’ kind of situation.” (A2).

However, some participants in our sample felt they did not have this acknowledgement and instead things were ‘glossed over’ and some experienced individuals and organisations refusing to accept responsibility.

“There was never really a kind of an actual acknowledgment at that time that oh that shouldn’t have happened. Or you know it was more just this kind of gloss over, ‘oh we understand your concern’, or ‘we’ll take that onboard’… not an actual like ‘that should not have happened’. Or ‘we’re sorry that happened’.” (F1).

Participants who were interviewed all acknowledged that mistakes happen, and accepted that, but it was when there was a sense of evasiveness or reluctance to acknowledge and apologise, that they felt further harmed. In the next patient’s story, she described it as a battle to finally get this acknowledgement and apology after a diagnosis that was missed many times. She felt she had to go the legal route to ‘chase’ the acknowledgement of the harm that was done to her. She spoke about on-going emotional trauma that took a physical and psychological toll on her, as she sought the acknowledgement and apology she wanted from the service.

“The whole process for me was a fight and a battle. But in the end, I got my apology so I did, I asked for my apology. That’s all I wanted, an acknowledgement and an apology. And I got an apology… It should have come a lot earlier, I shouldn’t have to chase, chase it.” (P1).

When this participant did receive her apology, she described it as a relief. An advocate also described this impact of acknowledgement as “a weight lifted off them” (A4).

“And that was a huge thing for me to be able to get an acknowledgement of the harm and the damage and the wrong that’s been caused to me.” (P1).

Participants also described experiences of apology during OD. In one instance of a low level of harm/risk, this participant recalled the immediate acknowledgement and acceptance of responsibility and how this endowed trust in the healthcare staff member.

“I completely trusted him, and I actually ended up bringing her back [*her daughter*] again for further, you know, I completely trusted him. And you know mistakes do happen and in a busy environment. But I just think if you just put your hand up and say you know ‘I’m sorry’, that’s all you wanted to hear really.” (F2).

#### Importance of clear and compassionate communication

While participants reported conversations about care experiences happening formally in meeting rooms and informally at the bedside, one issue that came up consistently was clarity regarding whether OD was happening or not. Staff, patients, and most frequently advocates, described the perceived reluctance to use the term ‘open disclosure’ and as a result, there was a lack of clarity about the process, next steps and often, this created confusion. One advocate recounted the story of being with a family and seeking clarity. She directly asked a consultant if their current meeting was an OD meeting, and he replied, “All my meetings are OD meetings….” which may demonstrate a lack of understanding on his part, but this response failed to give the family the clarity they were looking for regarding the process and next steps. Patient advocates, speaking to their experience supporting many families through the process, commented that the term ‘open disclosure’ is “skirted around” (A1), potentially due to fear of litigation.

“I suppose my biggest concern and issue in open disclosure is that the words open disclosure are rarely used…So they will use kind of other phrases like ‘would you like to come in to meet with the consultant’, ‘would you like to have a debrief’ or a ‘family meeting’ or a ‘review as to what has happened’. So sometimes it’s actually very unclear whether the person has actually had an open disclosure meeting or not.” (A1).

One factor that participants mentioned repeatedly was frustration at feeling staff were ill-informed or unprepared for OD meetings, leading to poor or conflicting communication between different healthcare staff and families. This emphasised the need for a team-based approach to planning and organising the meeting, ensuring the healthcare team was clear on knowing what they could speak to and clear on what details were still unknown about the event. Furthermore, effective planning about who will communicate with families and patients was considered critical. One story recounted by a healthcare professional reflects on the impact on trust between staff and families when this does not happen. Without all the available information, healthcare staff became defensive, and the family felt the truth was being withheld from them.

“The consultant knew there was a need for an open disclosure meeting and I felt a bit uneasy going into the meeting without having all the evidence before me because we had a lot of work that had been completed from a nursing perspective but really I was missing some really key bits of information from my medical colleagues… it became a bit of a car crash open disclosure meeting… the family were very disappointed about that, they really felt that that could have been managed completely differently.” (HCP1).

Sensitivity of communication was identified by many participants as crucial to the person who had experienced harm feeling listened to in the process. Again, the impact of poor communication from healthcare professionals in what was likely intended as an effort to reassure and console those who had experienced harm. However, rather than console, the language used sometimes angered people.

“It felt like, they had come to diminish my whole experience actually, and yeah it just, the, they just really played down the severity of the whole thing.” (P2).

“I suppose the only other thing, I suppose because it’s another one of my bugbears was, you know that I kept getting told all the way through ‘you’re fine’. ‘It’ll be fine, it’ll be fine’ and I asked like, I was in a group, I just said, I said ‘You say to someone it’s going to be fine’, I said ‘how do you define what’s fine for me?’ I said fine for me was not living this life that I’m living, that’s not fine for me. I’d have chosen death.” (P3).

#### Openness and sharing as a two-way process

When OD meetings took place, what participants valued was openness and transparency. Even when the news was distressing, upsetting and even frustrating, participants recognised and appreciated staff’s genuine attempts to be honest and open about what had transpired.

“You know we were saying to her that you’re really probably unwell because this has happened. We’re sorry, there was no words that we felt we could say, only to explain and tell the truth and you know she got up and shook our hands and said ‘thank you for telling me the truth’. I mean that’s fundamentally what it’s about.” (HCP2).

“I actually appreciated, if I’m honest, I remember back at the time, I appreciated their honesty. I was a bit annoyed, but I appreciated their honesty.” (F3).

Conversely, the deleterious impact on trust was evident when there was not this openness and transparency. In one case, information was promised to this individual about their experience of harm and was then subsequently withheld. Not only did this impact the relationship with healthcare providers, but it was also described as compounding the harm of the incident and delaying the person’s ability to process and manage the trauma of their experience.

“Any information you have, just give it to them they are going to, the patient is going to get it anyway. So just you have the information, that just feeds their mistrust, and it actually delays the trauma process because you are trying to get your head around it, you are missing a few pieces and then somebody says no you can’t have it. And it just makes things much worse.” (P2).

The opportunity for patients or relevant others to share their experience of care and the aftermath of when something goes wrong during that care was recognised as a crucial part in the process. One healthcare professional clearly explained why this was central to the process:

“That they get to tell their story, [it’s a] basic human right that they’re heard and understood… really you have to let people tell their story…. that’s the key to that connection” (HCP5).

However, not all participants were afforded this opportunity. In this patient’s story, she described how she felt she was dragging herself in for the meeting, even though she was still unwell and recovering. For days before, she was trying to decide how best to answer when the team asked her how she had been. However, she was taken aback when nobody asked.

“I was really shocked because the whole way throughout, you know, because I had questions of ‘what will I say when they ask me how I was?’. Because I’m trying to be strong, and advocate and fight for this cause. But also I have really suffered so it’s hard to be like ‘I didn’t want to go in [to work]’, ‘well I can’t do A, B, C, D’… So, nobody asked how I felt. That was appalling.” (P2).

#### Experiences of healthcare staff as patients in open disclosure

One notable finding in the data was the feelings of betrayal by their colleagues and peers and disillusionment with their organisation among healthcare professionals when they themselves experienced harm during healthcare, either directly themselves or through the experience of a loved one and had a negative experience of OD. One participant described her own experience of serious harm.

“I felt totally and utterly helpless, frustrated, I was actually, I took it really personally I was really hurt you know. Maybe I thought I was special you know because I was one of them” (P2).

However, other participants working in healthcare saw their training and experience as an asset when navigating the healthcare system for themselves or their families. It was felt that those without the same experience may not know how to pursue concerns, would not necessarily be aware of what quality care should look like or would not know about ODs processes.

“I think in some ways there’s positives in that if you have a healthcare background you maybe are more informed as to what good care should be. What you should expect, do you know what I mean?” (F1).

#### Lasting impact of negative experiences and open disclosure

Among participants who described a poor OD experience, or those who had to actively pursue the organisation to get an apology and acknowledgement of harm, it was apparent that this experience not only affected their own healthcare seeking and trust in the system, but also that of close family members long after the incident/experience had occurred. One participant described it as a generational trauma, where the experience impacted the whole family’s interaction with the healthcare system.

“I don’t trust the system now. I don’t trust my health care now and I’m very suspicious about my health care. Any time I have a test, any time I go to the GP, I ask for all my records” (P1).

“I used to keep going in because I was afraid they’d kill him. And I wanted to be there, every time they went to do anything I wanted to be there… My whole family were paranoid” (F3).

One advocate reflected on working with families over time as they worked through the OD process and seeing the impact it had on them when the process was not going well.

“You know the absolute trauma and anger and upset when they initially contacted our service and then like a year and a half two years down the line they are different people. You know, because I suppose they have changed because of what has happened. But not only that I suppose they’ve had that time to maybe not, that anyone ever you know not that anyone would ever be the same after something, after a terrible incident, but like they have changed.” (A4).

A common pattern also evident within this theme was the impact of poor experiences of OD on staff. One advocate recognised that there can be “trauma on both sides” (A4). Healthcare professionals described it as an intimidating process that provokes anxiety and fear. The quote below from a healthcare staff participant described their perception of what it means to be involved in OD and how it can indicate a culture of fear among staff.

**“**It can be an intimidating process when you have someone at a very senior level looking for core information, that’s hard. I can remember that particular nurse writing to me to let me know how she felt very unsupported by management and that she thought it was scapegoating her. And that was difficult because it’s, it’s not easy, it’s hard to, of course it made you reflect on the impact of these things happening, on the impact that these things can have on staff.” (HCP1).

## Discussion

This qualitative study aimed to explore experiences of OD from the perspective of those involved: patients, families and relevant others, healthcare staff and patient advocates. This analysis presented five key themes that were evident from the emotive stories and experiences recounted by participants: (i) The necessity of acknowledgement and apology, (ii) Importance of clear and compassionate communication, (iii) Openness and sharing as a two-way process, (iv) Experiences of healthcare staff as patients in OD, and (v) The lasting impact of poor experiences of OD.

From our analysis, it is evident that first and foremost, it is crucial for patients to receive an acknowledgement of harm and a genuine and sincere apology. This was very meaningful to all patients and family members interviewed and even those who were receiving life-changing news, the genuine apology was valued and appreciated. Those who did not receive this found it challenging to move on or reported ‘chasing’ this acknowledgement until they got it. As part of the process, transparency and honesty are key to building and maintaining trust between healthcare staff and patients/families and their relevant others. When this trust was broken, it was very difficult to rebuild. It is particularly fragile around the time that something goes wrong in healthcare and as such, Wu referred to this period as the ‘Golden Moment’ to honestly disclose what has happened to the patient or family “before patient trust erodes, and judgments harden” (p.179) [31]. A timely disclosure is valued and expected by family and patients and as described by Wu [31], can cause irreparable damage when this does not happen.

The importance of clear and compassionate communication has been emphasised in almost every previous study of this nature. However, perhaps unique to the Irish context is the widely reported reluctance to use the term ‘open disclosure’ when discussing issues with patients, families and advocates. As previous research has suggested, this is likely due to a fear of litigation, fear that being associated with OD means healthcare staff have made a mistake and/or the existence of a blame culture within organisations [11, 32]. Research in the Irish context from 2012 reported that staff felt they were “on trial” during OD process [32]. Given the current findings and the description of the fear and ‘trauma’ experienced by staff involved in OD, it is not clear if this perception has shifted in a major way in the almost 15 years since the previous study.

Participants from all perspectives in the current study (patients, advocates and healthcare staff) acknowledged the importance and value of creating space during formal meetings to hear the experiences of those who have been impacted by the patient safety incident. This echoes previous research which found that patients and families expect the OD process should be characterised by a responsive and reciprocal dialogue [13, 33]. Furthermore, the language we use when communicating about harm must be considered carefully, as what may be intended as efforts to reassure or console patients/families can be interpreted as efforts to minimise or dismiss patient experiences of harm. This has important implications for how staff are trained and how they convey empathy without minimising the person’s experience. This has led to suggestions for tailored, person-centred communication strategies that aligns with the individual’s preferences [34]. Researchers have advocated for active engagement of patients and on-going dialogue after the incident to ensure the organisation is meeting the patient’s needs [34].

A novel finding in this research is the experience of those working in healthcare and the additional layer of complexities when colleagues fail to be transparent and open about incidents when healthcare staff are patients in the system. This was described as akin to a double trauma by participants. Not only did they experience the harm and resulting negative experience of OD as a patient themselves, but they felt disillusioned, let down, unsupported and betrayed by their own peers. Given the high proportion of the population working in healthcare services in Ireland, it is not surprising that healthcare staff themselves are experiencing harm as patients themselves. What warrants further investigation is the impact this has on people returning back to work into a system when OD was not practiced by their colleagues. This appears to be a major gap in the evidence base currently.

It is evident from this research that compounded harm is happening through poor understanding and implementation of OD, and this is avoidable. The final theme reflected on the on-going impact of experiences of OD on subsequent engagement with the health service, trust in healthcare professionals and in the system and this impact can extend through the network of the individuals who has directly experienced harm. Ottomen and colleagues [35] explored the long-term impacts reported by patients and family members who experienced harmful medical events and outline the varied psychological, social, physical and financial impacts that may persist long after the patient safety incident has occurred. Loss of trust, grief-like experiences, anger and psychological well-being aspects were also evident in our study. This works speaks to the importance of researchers and healthcare systems attending to the long-term impacts of these incidents on patients and families.

What is evident is that due to a lack of research in the national context, we do not currently have a clear sense if the OD process is meeting the needs of patients, families and relevant others. While a limitation of this research is that it included a relatively small sample, this research provides evidence of inconsistent and unsatisfactory practices and therefore, ongoing training, monitoring and feedback mechanisms are needed to continuously improve and bring greater quality and consistency to the OD. Another limitation of this work was the unbalanced participant sample, as women represented the majority of respondents. According to the WHO, women make up 67% of the health and care workforce [36], and this may partially account for the overrepresentation of women in the advocates and healthcare staff samples. The low levels of male respondents in the patient/family sample may be due partially due to cultural norms regarding masculinity, health and discussing personal feelings and experiences. While there is evidence of changing norms regarding masculinity in Ireland, this may still be a factor [37]. Finally, the use of gatekeepers to recruit participants may have introduced bias to sampling. However, this was the approach that was deemed most appropriate and sensitive by our advisory group, which included patient/public partners.

The findings of this work suggest a need for the ongoing promotion and fostering of an open patient safety culture across the health system. Healthcare staff still have a considerable fear of litigation and of media attention when things go wrong in healthcare [1, 32, 38]. This fear presents a significant barrier to OD and staff stories from this research highlighted how pervasive this fear culture is. Promoting a safety and learning culture requires role modelling and supporting staff when incidents do occur, recognising that system failings can occur involving staff, but it is how its managed afterwards that can partly determine and mitigate the impact [39]. A team-based approach to OD would ensure that staff work together to prepare for OD meetings [34], there is clarity about which team member can speak to various aspects of care and the incident, and all team members provide a clear, honest and comprehensive account of what happened to the patient or their relevant other. Where it is appropriate, the involvement of junior staff for the purposes of learning should be encouraged, to normalise and de-stigmatise the process when things do go wrong. The importance of role modelling behaviours [40] has been highlighted by previous research and it is important that this is prioritised. Since this study was conducted, the Patient Safety (Notifiable Incidents and Open Disclosure) Act 2023 came into effect, which seeks to strengthen openness and transparency throughout the Irish health care system and mandates a legal requirement for OD in certain types of patient safety incidents. Further research on the impact of this change is required to understand if (and how) this may have affected disclosure processes and conversations. We echo the call made by Heyhoe and colleagues [41] for researchers to recognise and explore the “powerful influence of emotion when exploring patient safety problems” (p. 56). The highly emotive OD process for patients, families and healthcare staff necessities further work to explore the impact of the process on all stakeholders. It is only with this understanding can we hope to enhance and re-design processes and supports that meets the needs of those involved.

## Supplementary Information

Supplementary Material 1.

## Data Availability

Due to the sensitive nature of the research supporting data is not publicly available beyond the data reported in this paper. Anonymised quotes supporting this research are available from the lead author (ADB) upon reasonable request.

## Footnotes

1 According to the Patient Safety (Notifiable Incidents and Open Disclosure) Act 2023 in Ireland, a relevant person, in relation to a patient, means a person whom the patient has nominated for the purposes of this Act to attend meetings and assist the patient.
