# Supplementary material for: Understanding what shapes patient and family experience of the open disclosure process in the Irish healthcare context: a qualitative study
Source: BMC Med Ethics. 2026 Mar 11;27:80. doi: 10.1186/s12910-026-01436-0 (PMC13088738; doi:10.1186/s12910-026-01436-0)
Supplement: Supplementary file 1 — Supplementary Material 1. [file 12910_2026_1436_MOESM1_ESM.docx]

**Interview Topic Guide for Patients/Other relevant persons**

**Introduction**

Introduce self

Thank participant and their support person as appropriate for their time and participation. Express sympathy that they have had this experience and thank for being willing to share their story with aim of improving process for others in future.

Explain purpose of the interview, review the Participant Information Sheet with them to ensure their full understanding.

Ask what questions they have and answer them.

Request permission to audio-record interview.

Explain right to not answer any questions, pause, and withdraw at any stage.

Explain right to receive a copy of transcription.

**Background**

Can you tell me about yourself?

Thank you for taking part today. We are doing this research because we are interested in understanding the experiences of people who have been through the Open Disclosure process. We are hoping that by better understanding what the important aspects were to patients and relevant others, and more about their perspectives on the process, we hope to learn how we can best improve the Open Disclosure process for those who will be part of the process in the future. Thank you for agreeing to share your experiences of the process. You do not have to share all details of your experience with us if you do not wish to, rather we would like to learn from your experience to understand what parts of the process are particularly important. We hope this will inform how we get routine feedback from people who go through the process.

**Experience**

Can you talk me through what happened when you were told that something when wrong during your care? It might be easiest to tell the story from the beginning.

- What was the reason you were accessing the health service when something went wrong?
- Can you explain what happened that went wrong?
- When were you first told about it? Who told you about what happened?
- Where did this take place and what information was given to you?
- How did you feel?
- Who was involved with this process to support you?
- What were your interactions like with your healthcare team during and after what happened?
- What information were you given and how? (Formal/informal, in writing, many/few meetings?)
- What were your expectations in relation to open disclosure before you attended a meeting?
- Did you feel prepared to take part in the process? Did you feel you had enough/understandable information?
- What was your experience? (apology? Enough time? Sufficient support through process? Did you feel listened to?)

Were you supported to manage this news, if so how? Were you given what you needed? Was there anything else you felt was important but was missing?

How did the open disclosure process impact on you do you think? (and on relevant others that supported you during the process, if applicable)

At the end of the process, were you satisfied or dissatisfied? What makes you say this?

**Improvements**

What worked well or not well?

What do you think could be improved?

Was there anything you appreciated about the process, or you found particularly useful?

What was the most important part of the process for you? Why?

What do you feel is most important to other people who go through this process?

**Conclusions**

Is there anything else you would like to add about your experience that I haven’t specifically asked about?

Are there any concluding remarks/key messages you want to share?

Thank participant for their time and participation. Express sympathy that they’ve had this experience and thank for being willing to share their story with aim of improving process for others in future.

**Topic Guide for Healthcare Staff/Advocates**

**Introduction**

Introduce self

Explain purpose of the interview, ensure participant has read information sheet.

Ask if they have any questions or concerns and answer any questions they have.

Request permission to audio-record interview.

Explain right to not answer any questions, pause, withdraw at any stage.

Explain right to receive a copy of transcription.

Thank you for agreeing to share your experiences of the process. You do not have to share all details of your experience with us if you do not wish to, rather we would like to learn from your experience to understand what parts of the process are particularly important. We hope this will inform how we get routine feedback from people who go through the process.

**Background**

Can you tell me about yourself?

Have you had much experience working in health services in Ireland?

What does open disclosure mean to you?

**Experience**

Can you talk me through what happened when you realised something went wrong for a patient when under your care / when you supported a patient/relevant other through the open disclosure process? It might be easiest to tell the story from the beginning.

- Can you explain what happened that went wrong?
- How did you feel when you first realised what happened?
- What steps were taken?
- What were your interactions like with the patient/their relevant other/your colleagues during and after the event?
- What supports were you given to help you with the process?
- Did you feel you had enough/understandable information? Did you feel the patient/relevant other had enough information?
- What are your key reflections on this experience?

**Improvements**

What worked well or not well?

What do you think could be improved? Why do you say this?

Do you think the process achieved its objectives?

Was there anything you appreciated about the process, or you found particularly useful?

What was the most important part of the process for you? Why?

What do you feel is most important to other people who go through this process?

What do you feel is the most important part of the process for patients? What shapes their experience of the process?

What part of the process should we be getting patient feedback on?

**Conclusions**

Is there anything else you would like to add about your experience that I haven’t asked about?

Are there any concluding remarks/key messages you want to share?

Are you interested in continuing your involvement in the research project? Explain co-design WP3.
